# Trust Beyond Borders: European External Regulatory Influence on Access to Medicines

**DOI:** 10.1017/jme.2025.10140

**Published:** 2025

**Authors:** Pramiti Parwani, Katrina Perehudoff, Anniek de Ruijter

**Affiliations:** 1 University of Amsterdam, Amsterdam Law School, Law for Health and Life, Netherlands; 2 Amsterdam Institute for Global Health and Development, Netherlands; 3 Amsterdam Centre for European Law and Governance, University of Amsterdam, Netherlands

**Keywords:** European Regulatory Bodies, Pharmaceuticals Access, European Medicines Agency (EMA), European Patent Office (EPO), Trust

## Abstract

European institutions are widely recognized as wielding regulatory power in a globalized market, exporting its standards across borders and between sectors. This paper asks what institutional dynamics catalyze European external regulatory impact on pharmaceutical governance in low- and middle-income countries (LMICs). The research focuses on two European regulatory bodies, the European Medicines Agency (EMA) and the European Patent Office (EPO), and explores the dynamics of their technocratic outreach beyond European borders. We find that trust is a key underlying institutional dynamic facilitating some forms of European external relations. The agencies extend their influence through technical assistance, collaboration, and work-sharing with LMIC regulators, fostering a one-sided relationship of “technocratic trust.” This trust, reinforced by international regulatory frameworks that position the EMA and EPO as “trustworthy” regulators, enables these agencies to expand their regulatory influence beyond Europe. By critically examining the impact of this trust-building on LMICs’ regulatory autonomy, this research contributes to the broader discourse on European regulatory power in global health governance and highlights potential implications for pharmaceutical markets and access in LMICs.

## I. Introduction

The suspension of unsafe and/or ineffective medicines from the market, as was the case, for instance, with Vioxx^1^ and Trovafloxacin,^2^ illustrates the critical importance of medicines regulatory agencies for *protecting* public health.^3^ Beyond protection, regulatory actors including patent offices also have a key gatekeeping role in *promoting* access to medicines, i.e., by applying domestic patent law to determine which products are eligible for patent protection.^4^ For instance, the decision by the Indian Patent Office to reject a patent application for an HIV/AIDS medicine under Indian patent law meant that cheaper generic versions could be made available for patients not only in India, but also for export to other low- and middle-income countries (LMICs).^5^ Both types of regulatory bodies — medicines regulatory agencies and patent offices — share the common responsibility of providing timely and technical evaluations for the legal sale and commercial protection of pharmaceutical products, respectively. In other words, patent offices and medicines regulators are critical gatekeepers in a medicine’s lifecycle from bench to patient. The speed and accuracy of these regulators’ decisions not only opens markets for the producers of these medicines, but also profoundly impacts patient outcomes and public health. For instance, COVID-19 vaccines were approved for emergency use, and rapidly made available on the market due to expedited rolling reviews of preliminary data by medicines regulatory agencies, including the European Medicines Agency (EMA) and US Food and Drug Administration (US FDA).^6^

A longstanding challenge for these regulatory bodies, particularly those operating in resource-constrained LMIC settings, is conducting comprehensive product evaluations in a timely manner. Resource limitations, along with considerations of time and efficiency, are often cited as key reasons for promoting reliance and work-sharing arrangements between regulators.^7^ For instance, in the context of marketing authorizations for pharmaceutical products, the World Health Organization (WHO) promotes regulatory reliance as “a smarter, more efficient way” of regulating medical products, to improve access to safe and effective medical products.^8^ In the case of patent protection, the Patent Cooperation Treaty administered by World Intellectual Property Organization (WIPO) facilitates cooperation between patent offices by providing preliminary examination reports that national or regional offices can rely upon when deciding whether to grant patents.^9^

The movement towards regulatory reliance reflects a broader trend towards “regulatory globalization” — defined as the “process by which regulatory agencies extend their reach internationally”^10^ — with regulatory bodies being increasingly seen as intrinsically networked and interconnected across jurisdictions.^11^ Regulatory agencies extend their reach internationally, e.g., through informal arrangements, formal coordination and recognition agreements, and transnational networks amongst regulators.

Based on our findings, we put forward that the social practice of institutional trust-building is a crucial component to facilitate reliance amongst regulators. Based on evidence of the external influence of European regulatory bodies, we build on concepts from organizational and management sciences on interorganizational trust, to provide a theoretical framework to explain how European bodies — in particular here, the EMA and the European Patent Office (EPO) — are able to build trust among regulatory bodies in third countries beyond EPO and EMA Member States. In this context, trust is commonly defined as “… the willingness of a party to be vulnerable to the actions of another party based on the expectation that the other will perform a particular action important to the trustor, irrespective of the ability to monitor or control that other party.”^12^

We argue that sustained interactions amongst regulatory bodies in different jurisdictions — through joint working groups, provision of technical assistance, and participation in transnational regulatory networks — allow for certain regulatory bodies to build a reputation as credible and “trustworthy” regulators. We further contend that this trust mostly flows in a unidirectional way — with LMIC regulatory bodies in the Global South placing trust in and relying upon assessments and decisions by regulatory bodies in the Global North, usually without reciprocal recognition. Finally, we examine the effects of this unidirectional trust on pharmaceutical markets in LMICs, and consequently on access to medicines for the people there.

This paper specifically examines the external influence of two European regulatory bodies — the EMA and the EPO — as prominent examples of regulatory influence originating from Global North authorities. These organizations shape access to pharmaceuticals within their jurisdictions, as well as in other jurisdictions. While our analysis is focused on these two European regulatory bodies, the proposed framework is broadly applicable and can be extended to examine regulatory influence and trust-building by other Global North authorities.

Building on insights from law, European studies, and management and organizational sciences, we can gain understanding of some of the factors contributing to trust-building in global health regulation and gain fresh insight into the factors influencing equitable access to medicines worldwide. Our analysis shows that in combination — due to the intrinsic characteristics of these bodies, their sustained technical outreach activities, and international mechanisms which reinforce trust within them — the EMA and EPO are able to establish themselves internationally as trustworthy regulators. This trust facilitates reliance and recognition of their decisions, thereby not only expanding the influence of European regulatory standards but also the global *application* of these standards.

The next section provides a theoretical framework, combining previous research on norm dissemination through technocratic outreach activities and external trust-building by European regulatory bodies with a framework of interorganizational trust drawn from organizational sciences. Sections III and IV then look at how the EPO and EMA can cultivate the trust of their regulatory counterparts in other LMIC jurisdictions, thereby expanding the external influence of these European regulators and facilitating reliance upon their decisions by regulators in third countries. These sections also show how these reliance linkages shape pharmaceutical markets in third countries. Section V then discusses governance and policy implications arising from this reliance and articulates some conclusions.

## II. Inter-Institutional Trust Building Between Regulators

The first subsection outlines the mechanisms of regulatory influence and highlights trust as a critical catalyst for rule diffusion, while the second explores the factors facilitating interinstitutional trust.

### A. Mechanisms Behind the EU’s Global Regulatory Reach

Within European studies, the proliferation of EU regulatory bodies, and specifically also the EMA, has been explained as a credible commitment of EU Member States to determining and implementing scientifically appropriate levels of risk regulation.^13^ Externally, the EU’s role as a global regulatory power is well-documented, with the EU unilaterally exporting its regulatory standards across different sectors to the rest of the world.^14^

Previous work studying the EU’s influence as a global regulatory power has identified different mechanisms through which the EU exerts regulatory influence beyond its borders. Lavenex distinguishes between two types of external relations through which the EU exports its regulations globally — first, through its traditional foreign policy channels, such as international law-making through treaties and agreements; and second, through the technocratic outreach of its regulatory agencies (labeled as functionalist extension).^15^ Lavenex argues that a significant part of the EU’s global regulatory influence occurs through this latter channel rather than traditional foreign diplomacy. EU agencies often participate in highly specialized transnational networks, which offer an avenue for collaboration, coordination, and knowledge sharing with counterparts in other nations.^16^ Functional extension by highly specialized technical agencies, according to Lavenex, relies on indirect mechanisms of rule diffusion, and can occur through four means — *learning, socialization, competition*, and *emulation.* ^17^

*Learning* occurs when there is a change in policy preference based on rationally observing others’ behavior, realizing the benefits of that behavior and thereby adjusting policy to it.^18^ Learning involves a process of observation, changing the underlying beliefs, and consequently adapting a policy based on the newly acquired insights.^19^ On the other hand, *socialization* is guided by a sense of what is considered “appropriate” and involves “a process of inducting actors into the norms and rules of a given community.”^20^ Both learning and socialization rely on trans-governmental networks amongst agencies. *Competition* involves utilitarian considerations and entails one actor adapting its behavior to avoid the negative externalities caused by another actor’s internal policies.^21^ In contrast, *emulation* involves alignment with another actor’s regulations because of a perception that they are normatively superior or legitimate.^22^ Rule transmission through competition and emulation are largely due to indirect socioeconomic dynamics between countries and regulators.^23^

Lavenex’s conceptualization of agency-specific technocratic outreach aligns with previous research on regulatory globalization^24^ which highlights “subtle forms of epistemic cooption” as a horizontal form of influence.^25^ This conception is consistent with a growing body of international legal literature, which contends that a comprehensive understanding of the practices and impact of international law requires an examination of “the way in which international law unfolds on the mundane and ‘everyday’ plane,”^26^ for instance through administrative procedures and technological and commercial practices.^27^

Within European studies, specific “organizational structural characteristics” of EU agencies have been highlighted as relevant in enabling external policy diffusion beyond the EU. These organizational characteristics include a specific underlying rationale and mandate, clearly delineated in the founding regulations of the agencies, their expertise and specialization, administrative and technical capacity, and financial and decision-making autonomy from political bodies.^28^

Implicit in these different means through which rule diffusion across international borders can take place is the fundamental role of trust. Within legal studies, Drahos, a leading scholar on cross-border regulatory influence, puts forward that sustained technical assistance activities by the EPO to patent offices in LMICs have played a pivotal role in fostering “technocratic trust.”^29^ He defines this as “the trust that individuals within systems place in the technical output of other systems.”^30^ Drahos labels this as an “impersonal” form of trust, where the trust is “based on a confidence in the reliable performance of a system, rather than individuals.”^31^

Drahos describes how technocratic trust develops as a result of sustained technical assistance initiatives undertaken by the EPO over a long period of time, which results in a “creeping lock-in of systems,”^32^ where long-term technical assistance programs and access to the EPO’s databases reinforces reliance on the EPO’s assessments and decisions.^33^ This allows the EPO to further promote more reliance on its work products through formalized channels. The technical assistance and work-sharing initiatives offered by the EPO are also integrative, in the sense that it allows the EPO to integrate more and more patent offices into a broader technocratic community of patent examiners around the world that have been trained by the EPO.^34^

Outside of legal disciplines, research on interorganizational trust seeks to explain why organizations, and the individuals within them, trust other organizations. Our framework combines these insights with scholarship from European studies on rule diffusion and Drahos’s work on technocratic trust. We seek to explain how European bodies — specifically, the EMA and the EPO — cultivate trust amongst third country regulatory bodies.

### B. Facilitators of Interorganizational Trust Building

There are a range of factors determining the level of trust that one organization places in another. Central to the definition of trust mentioned above are the notions of showing vulnerability, the expectations about the performance of another entity, and the inability to control or check on that entity.

Kroeger argues that trust can become institutionalized, with individual trust transcending interpersonal relationships to become “an attribute of the organization … subsequently feed[ing] into further trusting conduct of its members.”^35^ Thus, organizational trust is not merely the sum of individual trust held by members of the trusting organization; rather, it develops into a distinct and enduring form through path dependence, where previous trust-based interactions and institutionalized practices shape subsequent cooperation even among new members with no direct experience of the original interactions. Schilke and Cook suggest that one organization’s perceptions of another organization’s trustworthiness can be based upon prior interactions between the two organizations, the general reputation of the latter organization, and the institutional categories that the latter belongs to.^36^

A systematic review exploring trust across different organizational levels highlights two key dimensions emphasized in a majority of definitions of trust:^37^ first, *positive expectations about trustworthiness*, referring to perceptions about the intentions of the trustee, and their reliability; and second, *readiness to accept vulnerability*, referring to an intention to take risk, suspend uncertainty, and rely on the trustee.^38^ This review also identifies different antecedents of interorganizational trust including characteristics of the trustor and trustee, their networks, and communication processes.^39^ Relevant characteristics of the trustee include previous assistance provided by the trustee,^40^ quality assurance and competence,^41^ perceived inequity between the organizations in their relationship so far,^42^ and the integrity of the trustee organization.^43^ Shared characteristics amongst the trustor and trustee that facilitate trust-building include any previous relationship and collaboration amongst the organizations,^44^ expectations of a future relationship,^45^ similarity amongst the two organizations in terms of strategic alignment and organizational dimensions,^46^ mutual adaptation,^47^ and a common business understanding.^48^

Taken together, these factors facilitate the establishment and maintenance of interinstitutional trust, which we draw upon to explain how the EPO and the EMA cultivate unidirectional trust from LMIC regulatory bodies in their work products and decisions. In the subsequent sections, we argue that this trust stems from the characteristics intrinsic to the EPO and the EMA, the sustained relationships that these European regulators build with their counterparts in third countries, which is reinforced by international mechanisms that position them as trustworthy regulators. We further show that the relationship of trust facilitates reliance upon the work products of the EPO and the EMA, especially by their counterparts in LMICs. Finally, in the conclusion, we examine potential implications of such trust and reliance on pharmaceutical markets and access to medicines in LMICs.

## III. The European Patent Office

Intellectual property (IP) law illustrates how everyday regulatory practices shape norms, with patent offices functioning as key sites for the application and development of IP norms, beyond the framework of substantive treaties.^49^ While international treaties such as the Paris Convention, the Patent Cooperation Treaty, and the World Trade Organization Agreement on Trade-Related Aspects of Intellectual Property Rights (TRIPS Agreement) set broad normative rules, patents themselves are inherently territorial rights governed by the domestic laws of sovereign states.^50^ Patents are shaped by the decentralized interpretations and contestation of patent laws by states,^51^ including through the day-to-day practices of patent offices. For instance, while the TRIPS Agreement provides the criteria for patentability — novelty, inventive step, and industrial applicability — it does not further define these criteria.^52^ Countries are thus free to define these threshold criteria for themselves in their domestic laws and regulations, which are then applied by a domestic patent office. Accordingly, the result of a patent application may vary across countries for these reasons. Thus, the actual practice of IP law is deeply influenced by national patent offices, whose examination processes can significantly shape the legal landscape for patent rights.^53^

Patent office practices are evolving through increasing collaboration and administrative cooperation, forming a global web of networks between national offices.^54^ These collaborative efforts, often involving the automation and outsourcing of patent searches and examinations, can influence the standards applied during the patent review process. For instance, in pharmaceuticals, reliance on less stringent patentability criteria for secondary patents could result in granting (more) patents and thereby adversely affect access to lower-priced generic medicines.^55^ While normative initiatives aimed at harmonizing substantive patent laws have faced resistance, the administrative cooperation between patent offices has not always been subjected to similar scrutiny.^56^ This administrative cooperation has thus grown, quietly shaping patent norms through non-binding work-sharing arrangements. Over time, these collaborative practices may become de facto customary norms, further influencing the global IP landscape.^57^

The European Patent Organization, established by the European Patent Convention (EPC), is often recognized as “a striking example of a centralized patent process” within Europe.^58^ It includes all EU member states as well as several non-EU countries from the European region and plays a central role in patent regulation across Europe. It consists of two main bodies: the EPO, which serves as the executive body responsible for processing patent applications, and the Administrative Council, a supervisory body composed of representatives from all member states.

The EPO is responsible for assessing all patent applications submitted to it under the EPC — where it grants a “bundle of national patents”^59^ which must be validated separately in each designated EPC member state — and now, also under the EU-specific Unitary Patent System^60^ where the EPO is the designated administering body.^61^ While the EPO is institutionally separate from the EU, it has long been considered to exercise functions similar to EU regulatory agencies.^62^ All EU member states are part of the EPO, and the EU exercises informal influence on the EPO’s functioning — evident, for instance, from explicit reference to EU directives in specific EPO regulations.^63^ The connection between the EU and EPO has become further formalized since the advent of the EU Unitary Patent System.^64^ Accordingly, while the EPO is not per se an EU regulatory body, it nonetheless functions as a product of regulatory powers vested in it by individual EU member states and is therefore examined in this paper.

One of the EPO’s priorities is international cooperation with their counterparts beyond EU/EPC member states. Accordingly, the EPO’s recent Strategic Plans emphasize the goal of expanding the reach and impact of the European patent as a key objective.^65^ The 2023 Strategic Plan highlights the value of this expanding reach as follows:

*Through a single high-quality search and examination procedure, inventions can now be protected with patents obtained from the EPO in 44 countries. It is therefore of strategic interest that the EPO expand its coverage, through agreements, partnerships, technical co-operation and, importantly, by exporting converged European quality standards.* ^66^

This international cooperation is realized through formal mechanisms such as validation agreements and reinforced partnerships, as well as more informal channels through data exchanges, encouraging the use of EPO’s search and examination facilities and work-sharing schemes.^67^ These formal and informal mechanisms are interconnected: as Drahos conceptualized, a “steady drip-drip” of sustained informal interactions over time^68^ — through training provisions, technical assistance, and work-sharing programs — increases the likelihood of concluding formal agreements, such as validation agreements and reinforced partnership agreements.

Validation agreements offer the highest form of strategic cooperation between the European Patent Organization and non-member states. An applicant who has already been granted a patent by the EPO can simply seek to validate the patent without further (substantive) examination in a state which has a validation agreement with the European Patent Organization.^69^ At the moment, the European Patent Organization has concluded validation agreements with six states which are not parties to the European Patent Convention: Morocco, Moldova, Tunisia, Cambodia, Georgia, and Lao People’s Democratic Republic (PDR).^70^ It has recently signed another validation agreement with Costa Rica which has yet to come into force,^71^ and is currently negotiating similar validation agreements with other countries and regional patent offices.^72^

On the other hand, the reinforced partnership program requires the partner patent office to systematically reuse the EPO’s work in examining patent applications that have already been granted by the EPO. Thus, to the maximum extent practical under the legal framework applicable in the partner state, the partner patent office should by default reuse the EPO’s search and examination results, as well as other written opinions and the final decision.^73^ At the moment, there are thirteen reinforced partnership agreements in force: Argentina, Brazil, Chile, Colombia, Ethiopia, Indonesia, Malaysia, Mexico, Peru, Saudi Arabia, South Africa, Ukraine, and the African Regional Intellectual Property Organization (ARIPO), which has 22 members.^74^

Unlike more substantive patent norms negotiated in bilateral or multilateral treaties, these validation agreements and reinforced partnership agreements are concluded as administrative or technical arrangements.^75^ Their implementation typically does not necessitate changes to national law; it can often be achieved through administrative regulations or bylaws that patent offices are usually permitted to establish under the national patent law framework.^76^ As a result, barring some notable commentaries,^77^ these agreements usually escape the same scrutiny as international treaties with substantive patent norms, such as the TRIPS Agreement or free-trade agreements with IP provisions.

These cooperation activities play an important strategic role in furthering the interests of the users of the EPO.^78^ They benefit from a larger geographic reach, and in particular, based on validation agreements and reinforced partnerships, they can obtain patents in areas outside the scope of the European Patent Convention.^79^ This becomes particularly relevant when seeking to understand patenting activity in least developed countries (LDCs) and regional IP offices in Africa, where a vast majority of the patents in these offices are granted to foreign-origin applications.^80^

Validation agreements and reinforced partnership agreements are specifically significant in context of LDCs, which, under the TRIPS Agreement, are exempt from providing patents for pharmaceutical products until 2034, or until they graduate out of LDC status — whichever is earlier.^81^ The EPO currently has validation agreements in force with two LDCs — Cambodia and Lao PDR — and is negotiating a new validation agreement with Ethiopia, another LDC.^82^ Signing a validation agreement with the EPO without recognizing LDC transition periods or exempting pharmaceutical product patents could mean that despite the TRIPS transition period, pharmaceutical patents granted by the EPO could also extend to LDCs, upon an application by an EPO patent-holder.^83^

The validation agreement between Cambodia and the EPO acknowledges that pharmaceutical products are excluded from patentability under Cambodia’s domestic law as per the exemption under TRIPS.^84^ The application will be subsequently examined when Cambodia starts granting pharmaceutical patents in the future — after its graduation from LDC status in 2029,^85^ with reference to prior art that had been disclosed when the application was first filed.^86^ However, the validation agreement with Lao PDR — a country graduating from LDC status toward the end of 2026^87^ — does not have a similar exemption for pharmaceutical products.^88^

Furthermore, not all LDCs have adopted these TRIPS flexibilities into their national legislation, and implementing the TRIPS flexibilities after entering into a validation agreement with the EPO may be challenging.^89^ Moreover, in the coming years, as Lao PDR and Cambodia graduate out of LDC status and start implementing pharmaceutical patents, current technical assistance and cooperation agreements with the EPO risk creating a “creeping lock-in effect,” fostering reliance and path dependence that could complicate future policy adjustments.^76^

Reliance upon the EPO’s assessments and decision-making is facilitated by its reputation as a trustworthy patent office, even beyond its jurisdiction. A combination of factors facilitates this trust-building, and consequent reliance by other patent offices on the EPO’s patent reviews and final decisions. These factors, examined in more detail below, are analogous to those mentioned above in the theoretical framework on interorganizational trust in global health regulation in Section II.

*First*, and most relevantly, in line with what Drahos called a “steady drip-drip” of EPO’s technical assistance to patent offices in third countries, we see that this assistance helps in building technocratic trust in the EPO by third country patent officers, especially those in resource-constrained settings.^90^ Over time, sustained technical assistance activities and informal cooperation, such as through exchange of data, encouraging the use of EPO’s search and examination facilities, and work-sharing schemes allows the EPO to further push for more reliance on EPO’s work products through the formalized means of validation agreements and reinforced partnership agreements.

*Second*, this technical assistance is made possible because of the inherent characteristics of the EPO. For Drahos, it is the EPO’s strong financial position and autonomy over its considerable revenue — mainly from fees related to patent applications, filing and search, examination, opposition, appeal, and renewal — which allow it to carry out many long-term technical assistance programs.^91^ The EPO’s budget (2.57 billion euros in 2023) is fully funded by fees paid by its users who pay for the examination and maintenance of their patents.^92^

The EPO’s extensive digital, searchable patent database, Espacenet, is another inherent characteristic that enables foreign reliance. Espacenet is the “largest single source of technical information in the world … containing millions of patent documents.”^93^ It allows users to access patent applications filed before, and granted by, different patent offices, and has garnered a reputation as one of the most comprehensive in the world.^94^ Work sharing amongst patent offices is enhanced by patent offices being able to consult publicly available digital patent databases published by some patent offices. Furthermore, as Drahos described, when regulators from LMICs would visit EPO offices as part of trainings and technical assistance activities, the EPO’s digitized work systems stood out as particularly impressive and efficient, especially when contrasted against resource limitations faced by LMICs, thereby fostering EPO’s assessments and patentability decisions.^95^

*Third*, international reliance on the EPO’s reviews and decisions are further facilitated by the global cooperative mechanisms in which it participates, which also enhance its reputation as a trustworthy regulator. Under the World Intellectual Property’s Patent Cooperation Treaty (PCT), the EPO acts as one of the designated International Searching Authorities (ISA).^96^ There are currently 24 national and regional patent offices, including the EPO, which have been designated as ISA based on agreements with the WIPO.^97^ Most of these 24 patent offices are based in high-income and upper-middle-income countries, barring a few LMICs: India, Egypt, Philippines, and the Eurasian Patent Office, which includes two LMICs — the Kyrgyz Republic and Tajikistan.^98^

Applicants applying through the PCT can designate one of the ISAs to search for prior art and, if the applicant desires, the ISA will issue a preliminary international search report on the patentability of the product in light of prior art.^99^ A 2023 study that examined 4,884 patent applications filed in parallel at the EPO, the US Patent and Trademark Office, and the Japan Patent Office found that the EPO was designated as the ISA in 63% of the PCT applications.^100^ This means that the EPO usually becomes the first office to publish its search report for that application, and its reports are often relied upon by other patent offices, including those in high-income countries, such as the USA and Japan.

The discussion above shows that informal outreach and sustained technical assistance programs by the EPO allows it to cultivate “technocratic trust” in its assessments and decisions, eventually facilitating more formalized reliance upon its decisions through validation and reinforced partnership agreements with patent offices in third countries. This trust is further sustained through international mechanisms, such as the Patent Cooperation Treaty, which reinforces the credibility of the EPO as a trustworthy patent office.

Patent offices in other countries, especially those operating under resource-constrained settings, often rely upon the EPO’s evaluations and decisions — through formalized channels such as the PCT route where the EPO is often designated as an ISA, and bilateral cooperative arrangements such as validation agreements and reinforced partnership agreements, as well as informal practices.^101^ For instance, previous research on ARIPO — a regional IP office for anglophone African countries — highlights that ARIPO heavily relies on international search reports under the PCT route, as well as EPO guidelines.^102^

Reliance and other cooperative arrangements amongst patent offices in different jurisdictions undoubtedly offer many benefits through efficiency gains. It avoids duplication of work already conducted by the EPO, which is especially desirable for patent offices with scarce human and financial resources. Shorter evaluation timelines may also encourage more pharmaceutical companies to market their medicines in those countries.^103^ Further, work-sharing arrangements and joint assessments — if conducted in a supportive and sustainable manner — can support capacity-building, enhancing expertise and institutional development.

At the same time, we argue that reliance — especially when it is unidirectional, flowing only one way from a lower-resourced patent office to the EPO — also raises critical questions about the effective exercise of sovereignty by the relying patent office. Patents remain inherently territorial rights governed by domestic laws of each state,^104^ and formally the patent office retains sovereignty over its decision-making.^105^ However, resource constraints in LMICs and a push for efficiency maximization (e.g., emphasis on maximizing the number of patents granted and expediting the assessment time) can amplify reliance on the EPO, with a risk that this reliance reflects deference to the EPO’s reputation as a “trustworthy” patent office, rather than a critical evaluation of its decisions.^106^ This concern is further exacerbated when one considers that domestic legal frameworks and applicable patentability standards vary across countries — especially in LDCs which benefit from TRIPS exemption — as do public health needs. The EPO’s decisions are based upon relevant criteria under the European Patent Convention and may not be warranted under the domestic laws of third countries.

Therefore, while leveraging EPO assessments can offer pragmatic advantages, it is essential that such reliance remains context-sensitive, is critically evaluated, and balanced alongside interests of independent decision-making by third-country patent offices in line with applicable legal frameworks and public needs of the latter’s jurisdictions.

## IV. European Medicines Agency

The EMA was set up in 1993 through a Council Regulation, in the backdrop of the thalidomide tragedy, which highlighted that the single market must be accompanied by at least some degree of centralized pharmaceutical regulation at the European level.^107^ Responsible for the pre-market technical evaluation and post-market monitoring of medicinal products, the EMA offers a centralized pathway for granting a single marketing authorization valid throughout the EU and European Economic Area countries — Iceland, Liechtenstein and Norway.^108^ While this centralized pathway is available for all medicines, it is compulsory for certain classes of pharmaceuticals, including orphan medicines, advanced therapy — medicines and new active substances to treat HIV/AIDS, cancer, and diabetes.^109^ The EMA evaluates pharmaceutical licensing applications submitted to it and provides a recommendation to the European Commission, which then issues a legally binding decision based on that recommendation.

Although the EMA was established primarily to serve the EU’s internal market, it nonetheless plays a significant role in extending the EU’s external influence over third-country national regulatory authorities (NRAs). The EU Global Health Strategy envisages a role for the EMA in offering regulatory training with a view to enhancing public health capacities, especially in pharmaceutical medical devices and technologies.^110^ Similar to the EPO, the EMA also actively builds networks with third-country NRAs through its technocratic outreach activities — both bilaterally as well as through international regulatory pathways that will be described below. While the EMA has mutual recognition (i.e., bidirectional) agreements in place with counterpart NRAs in several high-income countries, regulatory reliance in the case of LMICs is often one-sided, with LMIC NRAs relying on EMA decisions, usually without reciprocal recognition for their own regulatory approvals.

To better understand the EMA’s external influence on third-country NRAs, it is essential to situate the EMA (and other NRAs) within the international regulatory environment in which they operate. While there is no overarching legal framework governing pharmaceutical regulation at the transnational level, policy guidelines adopted by international associations and the WHO offer instruction and guidance to regulatory agencies.^111^ Two international bodies play important guiding roles in global regulatory affairs.

First, the International Council for Harmonization of Technical Requirements for Pharmaceuticals for Human Use (ICH) is a public-private transnational regulatory network bringing together medicines regulatory agencies and the pharmaceutical industry to work towards greater harmonization of requirements for registration of pharmaceutical products and relevant guidelines.^112^ ICH guidelines are often adopted by national and regional regulatory authorities, including those that are not members of the ICH.^113^

As a founding member of the ICH, the European Commission (and by extension, the EMA as the responsible agency) is recognized as a Stringent Regulatory Authority (SRA), a status that — as will be discussed below — facilitates greater reliance upon the EMA’s decisions. Designation as an SRA is based purely upon initial membership of, or association with, the ICH, resulting in criticism about the democratic credentials of such designation due to concerns with transparency and exclusivity.^114^ To democratize this process, and expand the pool of “trustworthy” regulatory authorities, the WHO has in recent years introduced a new designation of WHO-Listed Authorities.^115^ This latter designation is based upon an assessment carried out by the WHO against the Global Benchmarking Tool, and is intended to replace reference to SRAs in WHO regulatory pathways such as the Prequalification Programme and Emergency Use Listing discussed below.^116^

The second international actor that is influential in providing technical advice for pharmaceutical regulation is the WHO itself; specifically, its departments and committees providing technical advice for pharmaceutical regulation are highly influential. For example, the WHO Expert Committee on Specifications for Pharmaceutical Preparations promotes regulatory reliance — defined as when a “regulatory authority in one jurisdiction takes into account and gives significant weight to assessments by another regulatory authority … in reaching its own decision … [while remaining] independent, responsible and accountable for the decisions taken…”^117^ as “a smarter, more efficient way” of regulating medical products, to improve access to safe and effective medical products.^118^ Regulatory reliance is mentioned as one of the Good Regulatory Practices highlighted by the WHO Expert Committee.^119^

**Figure 1.**
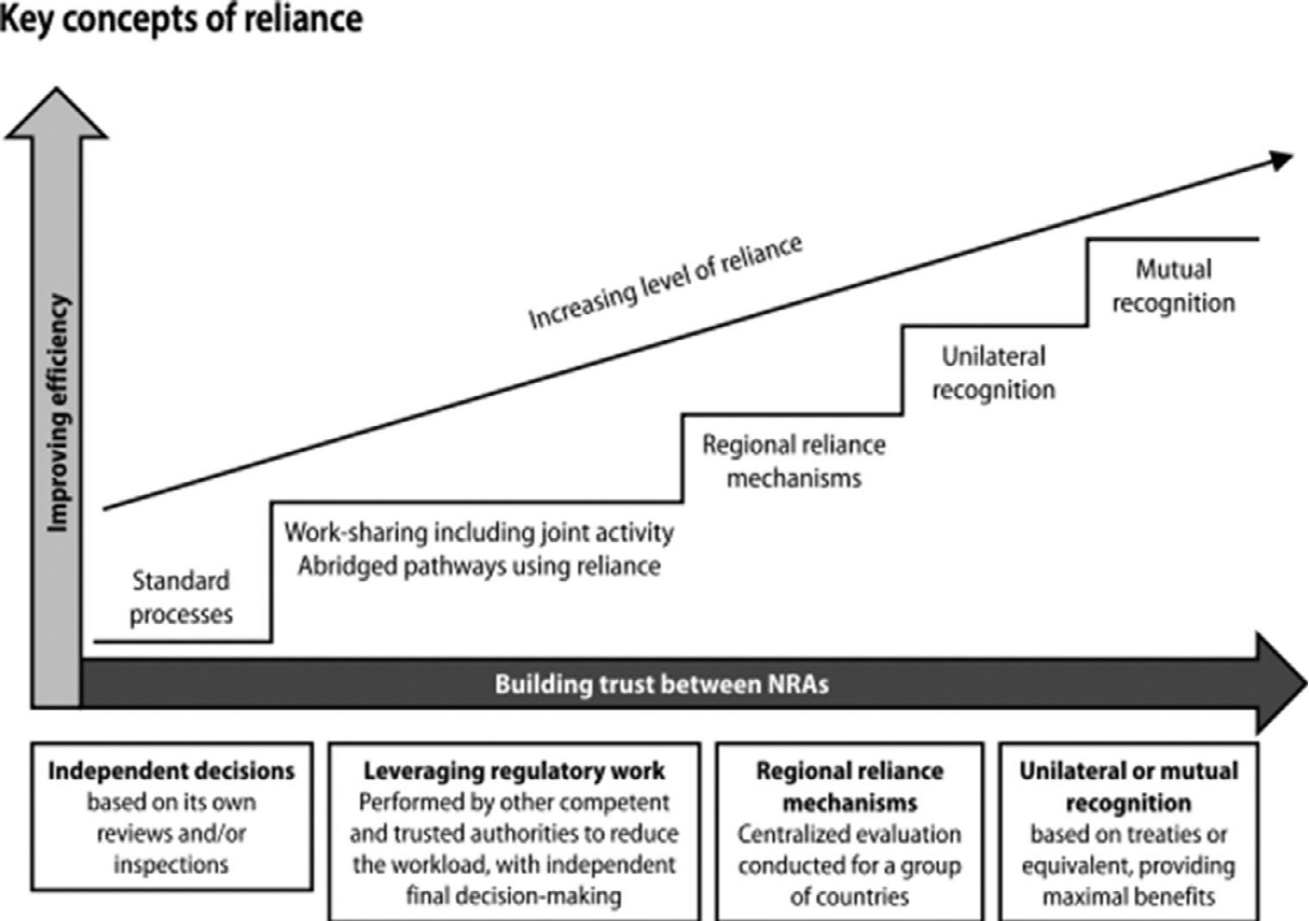
The role of trust in regulatory reliance. Source: WHO Expert Committee on Specifications for Pharmaceutical Preparations, *55th Report, Annex 10: Good reliance practices in the regulation of medical products: high level principles and considerations* at 245.

Moreover, the WHO Expert Committee highlights the crucial role of trust in regulatory reliance.^120^ Similar to Drahos’s conception, the WHO Expert Committee explains that trust usually grows as familiarity deepens (see Figure 1). As a regulatory authority gains more knowledge and understanding of another regulator’s work, it begins to place greater trust in it. Before a regulatory authority formally begins to rely on another authority’s output, there are usually pilot initiatives to build familiarity with, and increase confidence in, the other’s work.^121^ Facilitated by information exchanges and work-sharing arrangements, familiarity and trust build over time, and often lead to formalized reliance upon, and even recognition of, another regulatory authority’s decisions.^122^

The WHO Good Reliance Practices mention sovereignty of decision-making as one of the principles of good reliance practices, as it remains the prerogative of the NRA whether, and to what extent, it wants to rely on foreign regulatory systems.^123^ Thus, ostensibly, in choosing whether to rely on a foreign reference regulator, NRAs maintain independence and sovereignty over their decision-making. However, local resource constraints and the international regulatory approval pathways cultivated to ease the pressures of those constraints, reinforce trust in select regulatory authorities.^124^ In this context, questions are raised about to what extent LMIC NRAs can retain independent decision-making space when relying on Global North regulatory agencies positioned as trustworthy agencies.^125^

Regulatory reliance is also embedded within the EMA’s governing regulation, with Article 58 of Regulation (EC) No 726/2004 providing the EU-Medicines4All (EU-M4all) procedure to facilitate regulatory reliance upon the EMA’s positive opinions by third-country regulators.^126^ The EU-M4all program empowers the EMA to issue scientific opinions on pharmaceutical products for use only in countries outside of the EU, providing an assessment upon which regulatory authorities outside the EU — in practice, usually regulators from LMICs — may choose to rely.^127^

In line with Article 58 of Regulation (EC) 726/2004, the EU-M4all procedure is undertaken in cooperation with the WHO,^128^ and the assessment is collaborative, involving the WHO and regulators of countries intending to use the medicinal product.^129^ In this way, the EU-M4all procedure considers the suitability of the product in light of local circumstances in countries outside the EU. Regulators from non-EU countries are invited to participate in the assessment process, and they are provided with relevant training. Sustained interaction and training between the EMA and non-EU regulators help build trust over time, enhancing EMA’s regulatory reach and further building its credibility as a trustworthy regulator.^130^

NRAs in third countries may choose to rely solely upon the EMA’s opinion, or combine it with assessments from regulators in other countries or with international approvals granted by the WHO — for instance, through the WHO Prequalification and Emergency Use Listing mechanisms, as discussed below.^131^ Of the 10 positive EU-M4all opinions issued for new medicines between 2004–2019, 6 medicines were subsequently granted 138 regulatory approvals in 90 countries.^132^ Data limitations make it difficult to clearly state how much of this can be attributed to the EMA’s positive opinion.^133^

Beyond the formal means of the EU-M4all procedure, third-country NRAs often rely on the EMA’s regular product assessments and/or the European Commission’s central marketing authorization decisions for pharmaceutical products meant for the EU internal market.^134^ Unlike the EU-M4all procedure, the EMA’s central marketing authorizations are based on an assessment of the suitability of the product within EU borders, rather than conditions in third countries.^135^ Nonetheless, the EMA is often cited as a reference authority for pharmaceutical approvals in other countries. For instance, 13 regulatory authorities in the Latin American and Caribbean region explicitly recognize or otherwise abridge their local authorization procedure by referring to a prior approval by a reference regulator — including the EMA, US FDA and Health Canada.^136^ In several cases, these approvals based on reliance have been criticized for not considering the local public health priorities, and for being grounded in weak scientific evidence (which was the only evidence available at the time of the initial approval by the reference regulator).^137^ Some Latin American regulators that rely on EMA and FDA regulatory standards are also recognized as reference authorities for evaluating biosimilars within the region.^138^

This regulatory reliance upon the EMA is facilitated by a combination of factors. *First*, the EMA’s transparency with respect to its assessments is a major positive factor facilitating reliance upon its evaluations. The EMA makes public a European Public Assessment Report for each evaluated product (regardless of outcome) and other relevant information.^139^ It also proactively makes available the clinical data submitted to it in the course of pharmaceutical assessments, which not only allows use of this data for secondary research but also enables reliance upon the EMA’s decisions by other regulators. Between 2016 and April 2021, the EMA published clinical data for 123 pharmaceutical products — significantly higher than the US Food and Drug Administration and Health Canada.^140^

*Second*, sustained interactions and training between the EMA and non-EU regulators help build trust over time, enhancing EMA’s regulatory reach and further building its credibility as a trustworthy regulator.^141^ This is conceptually similar to Drahos’s notion of technocratic trust in the EPO: sustained technical assistance over time leads to increased familiarity with the EMA’s way of working and contributes to trust-building, eventually fostering reliance on the EPO (and as we argue, the EMA).

The crucial role of trust in regulatory reliance has been highlighted by the WHO Expert Committee on Specifications for Pharmaceutical Preparations.^142^ Similar to Drahos’s conception, according to the WHO Expert Committee, this trust usually grows as familiarity deepens (see Figure 1). As a regulatory authority gains more knowledge and understanding of another regulator’s work, it begins to place greater trust in it. Before a regulatory authority formally begins to rely on another authority’s output, there are usually pilot initiatives to build familiarity with, and increase confidence in, the other’s work.^143^ This occurs through information exchanges and work-sharing arrangements — which lead to increased familiarity and build trust over time, and often eventually lead to formalized reliance upon, and even recognition of, another regulatory authority’s decisions.^144^

In addition to technical outreach to third-country regulatory authorities carried out bilaterally or regionally by the EMA, we observe in these institutional practices that technocratic trust in the EMA is further built and sustained by its participation in international organizations and networks. Therefore, *third*, we contend that the reputation of the EMA as a “trustworthy” regulatory authority is further reinforced by its place in the international regulatory pathways maintained by the WHO — namely through the WHO Prequalification Programme, Collaborative Registration Procedure, the Emergency Use Listing — and the EMA’s longstanding status as an SRA.

The EMA’s evaluation procedures play an important role in the WHO Prequalification Programme. Medicines (excluding vaccines) that receive a positive evaluation under the EU-M4all procedure can be prequalified by the WHO without any further review.^145^ Prequalification of vaccines is based upon prior regulatory approval from a WHO-listed Authority, such as the EMA.^146^ In this way, the WHO Prequalification Programme amplifies the global influence of the EMA’s technical evaluations and the EU’s market approval decisions. For example, in a study of 21 NRAs across Africa, 95% of the participating NRAs reported that they relied on the WHO Prequalification Programme.^147^

Further, the EC’s status as an SRA permits the EMA to participate in the WHO-led SRA Collaborative Registration Procedure, where medicines approved by an SRA can then be submitted by the applicant to other NRAs with the same dossier.^148^ All participating NRAs have access to the full assessment done by the concerned SRA, but reliance by participating NRAs remains voluntary.^149^

During public health emergencies, the WHO Emergency Use Listing (EUL) is used to provide a one-year conditional authorization to unlicensed vaccine candidates based on initial data. Where the vaccine has been previously approved by an SRA (like the EMA), an abridged pathway is available for EUL designation.^150^ During the COVID-19 pandemic, the first vaccine to be granted EUL in December 2020 was based on the EMA’s conditional marketing authorization. Subsequently, nine African regulators relied solely on the WHO EUL to approve the vaccine domestically.^151^

Thus, international regulatory pathways play a key role in expanding the EMA’s reach globally. On the one hand, procurement agencies like Gavi and the United Nations Children’s Fund (UNICEF) rely on WHO Prequalification and EUL in choosing which medicines and vaccines are safe and of assured quality for international distribution.^152^ On the other hand, through the collaborative registration procedure, NRAs in LMICs rely on assessments and decisions by SRAs and the WHO Prequalification team. Given the WHO’s own reliance upon the EMA’s assessment, reliance by other NRAs on the WHO Prequalification and EUL further represents additional indirect, yet still very pertinent, effects of the EMA beyond its jurisdiction. Furthermore, the EMA’s designation as an SRA and a WHO-listed Authority also substantiates its credibility internationally as a trustworthy regulatory authority, thereby encouraging further regulatory reliance upon it.

## V. Discussion

Drawing from literature within organizational sciences on interorganizational trust and previous work on the external influence of EU institutions and bodies, we identified certain characteristics of two European regulatory bodies (EPO and EMA) that cultivate trust in their output and facilitate reliance by foreign regulatory bodies. The trust placed in the EPO and EMA by their counterparts in LMICs has a potential influence on which medicines are patent-protected and market-approved in LMICs, which in turn shapes access to these medicines for domestic populations.

With respect to antecedent factors facilitating interorganizational trust, we highlighted that inherent characteristics of the two European regulatory bodies positively affect trust by other bodies in their output. Both EMA and EPO make their assessment reports and reasoned decisions publicly available and thereby retrievable by foreign regulatory bodies. This enhances **transparency**, and provides a valuable source of information, especially in situations where regulatory bodies in other jurisdictions may be facing resource limitations. Transparency permits some evidence and decisions to be questioned and challenged, which, when done, allows for establishing some **quality assurance** and suggests a certain degree of competence in regulatory decision-making by the EPO and EMA. This therefore facilitates trust in the technical assessments of the EMA and EPO and thereby facilitates reliance on those assessments by their foreign regulatory counterparts.

Further, **previous relationships** between European regulators, on the one hand, and their regulatory counterparts in other jurisdictions, also foster trust. Contact amongst regulatory bodies in different jurisdictions may start off as informal interactions and work-sharing arrangements on a pilot basis. Over time, as a regulatory authority gains more knowledge and understanding of another regulator’s work, the former begins to place greater trust in the integrity of the latter. With increasing familiarity and trust, informal exchanges usually transition to more formalized reliance and recognition mechanisms. Especially in the case of LMICs, the EPO and EMA offer training and technical assistance activities that are critical vehicles for building trust and working towards reliance.

Most importantly, from a global health governance perspective, reliance upon the EPO and EMA is also facilitated by international regulatory pathways, in which certain regulatory authorities — usually from the Global North — are recognized as “trustworthy” regulators to be relied upon. For example, the Patent Cooperation Treaty encourages cooperation amongst patent offices to further streamline their work, with the EPO recognized as an International Searching Authority, upon whose reports and written opinions other patent offices may choose to rely upon. Similarly, WHO encourages regulatory reliance on specific agencies (designated as WHO-Listed Authorities, such as EMA) as good regulatory practice. Notably, regulatory bodies positioned as “trustworthy” by these international initiatives are almost always situated in high-income and upper-middle-income countries.

The designation of the EPO as an International Searching Authority, or of the European Medicines Regulatory Network (represented by EMA) as a WHO-listed Authority also means that these bodies cultivate a wide-reaching **reputation** as trustworthy regulatory bodies. This reputation can allow the EPO and the EMA to expand their reach beyond only the regulatory authorities that they had previously specifically cultivated a relationship with. As a result of reinforcement through international pathways, domestic regulatory bodies in other jurisdictions, especially LMICs, become more likely to accept the EPO and EMA as trustworthy regulators, and they may come to rely on their assessments.

The above-mentioned aspects — transparency, quality assurance, reputation, and previous relationship with patent offices and NRAs in third countries — are all factors which, as discussed in the theoretical framework, facilitate interorganizational trust-building and contribute to technocratic trust in the EPO and the EMA by third-country regulators.

## VI. Conclusion

Having highlighted the institutional dynamics that facilitate trust-building in the EPO and EMA by their regulatory counterparts in other jurisdictions, we now turn to the implications of such trust on pharmaceutical markets abroad, and consequently global access to medicines. While work-sharing and reliance are associated with several benefits arising from efficiency gains, some concerns also persist, especially where reliance is unidirectional, which, as the discussion in the previous section shows, it often is in the context of European regulatory bodies and their counterparts in LMICs.

Work-sharing arrangements and reliance amongst regulatory bodies (such as on the EPO and the EMA) undoubtedly come with efficiency gains. It prevents the duplication of work conducted by other bodies and can support abridged or accelerated reviews, which may be desirable benefits for agencies with limited human and financial resources. Expedited regulatory assessments of patentability or for marketing authorization may also allow medicines to reach certain markets faster, which can support access to medicines in that jurisdiction if they are affordable and available in sufficient quantity. At the same time, work-sharing arrangements and joint assessments can also lead to capacity-building within regulatory bodies with less experience, ultimately increasing their expertise and operational capacity if conducted in a supportive and sustainable manner.^153^

On the other hand, when trust and reliance are unidirectional (i.e., flowing from resource-constrained regulatory bodies to European regulators), concerns emerge regarding the **effective exercise of sovereignty and the capacity for independent, informed decision-making** by LMIC regulators who choose to rely on the assessments of the EMA and EPO. In principle, cooperation and reliance by two or more authorities often remain voluntary, with the designated regulatory authority remaining ultimately responsible for its decisions within its territory. Patents and market authorizations are always “territorial”^154^ and are limited to the jurisdiction where they were granted. Reliance by patent offices and NRAs on their counterparts in other jurisdictions remains voluntary, with each regulatory authority ultimately responsible for its decisions within its territory.

However, there are concerns that unidirectional reliance from a resource-constrained regulator upon a more well-resourced or renowned regulatory authority may largely be based on the latter’s reputation, without critically examining the quality of its output or its abilities.^155^ Moreover, when making decisions on patentability or marketing authorization within their own jurisdictions, the EPO and EMA do not consider the specific contexts of foreign countries whose regulators may later “copy” or rely on European regulatory decisions.^156^ Given the key differences amongst countries of the world in terms of applicable laws, health regulations, local resources, and economic and social conditions, unquestioning reliance on European decisions in a non-European context may carry substantial risks, with implications for access to medicines. For instance, if relying upon the EPO, another patent office grants or validates a patent where it is not supported by the applicable domestic law — such as in LDCs benefiting from the TRIPS transition period — this may impede access to more affordable generic products in that country. Similarly, as discussed above, whereas NRA blindly relies on another regulatory authority to grant approval for a medicine, questions remain about what happens when the original marketing authorization is subsequently revoked, especially due to safety or efficacy concerns.

The potential erosion of the capabilities of regulatory authorities in LMICs is made possible due to resource-constrained settings and a push for efficiency maximization (e.g., emphasis on the proportion of approvals granted as compared to the number of applications). This underscores the need for sustained independent regulatory capacity-building, especially in LMICs, and reliance pathways that consider contextual differences between nations that may affect the transplantation of one regulatory decision to another jurisdiction.^157^ This requires domestic and international investments in both financial and human resources in LMIC regulatory authorities and a rethinking of the utilitarian efficiency maximization approach.

A second aspect that requires further empirical research is the potential market-making effects of regulatory harmonization and reliance amongst patent offices and NRAs across jurisdictions, with a focus on identifying which pharmaceutical producers benefit from such reliance. We argue the EPO and EMA’s external regulatory influence primarily benefits its clients — the pharmaceutical companies which have previously been granted patents and marketing authorization by the EPO and EMA respectively — by streamlining market entry for these producers beyond only European markets. Especially where this reliance is unidirectional, as it often is in the case of LMIC regulators relying upon the EPO and EMA, that trust in and reliance upon the EPO and EMA allows their clients to bring their products to foreign markets sooner, thereby maximizing their opportunity to capture market share early in new markets.^158^ In this manner, European regulatory bodies may indirectly facilitate market penetration even in regions outside their formal jurisdiction.

More empirical research is needed to identify the origins of applicant pharmaceutical companies that apply for patents and marketing authorizations at the EPO and EMA and subsequently benefit from unidirectional regulatory reliance by patent offices and NRAs in LMICs. Understanding who gains from these reliance mechanisms is crucial to assessing their impact on pharmaceutical production capabilities and markets, and consequently on pharmaceutical access. We hypothesize that the primary beneficiaries are transnational pharmaceutical companies originating in Europe and other Global North countries. These firms are best positioned to capitalize on the unidirectional trust and reliance upon the EPO and EMA by LMIC regulators, leveraging such reliance to accelerate market entry and expand their commercial presence in LMICs, often with limited scrutiny or reevaluation at the local level.

In conclusion, while reliance on the EPO and EMA provides efficiency gains and facilitates speedier entry of medicines into new markets, a more balanced and context-sensitive approach is necessary to ensure that reliance does not undermine the local needs for pharmaceutical production and access to safe, effective, and affordable medicines in LMICs. Moving forward, there is a need for greater scrutiny and open dialogue around reliance mechanisms and capacity-building initiatives, with a focus on the impact on pharmaceutical producers and markets in LMICs and resulting implications for access to medicines. In parallel with reliance and regulatory harmonization, capacity-building efforts should also be diversified and strengthened through complementary approaches, including bolstering regional initiatives and South-South collaboration among NRAs and patent offices. Such efforts can empower LMIC regulators to exercise independent, contextually informed decision-making in line with their domestic legal frameworks and public health needs.
